# Potential Benefits of Physical Activity in MCI and Dementia

**DOI:** 10.1155/2020/7807856

**Published:** 2020-02-12

**Authors:** Hallie Nuzum, Ariana Stickel, Maria Corona, Michelle Zeller, Rebecca J. Melrose, Stacy Schantz Wilkins

**Affiliations:** ^1^VA Greater Los Angeles Medical Center, Los Angeles, CA, USA; ^2^Department of Psychology, University of Notre Dame, Notre Dame, IN, USA; ^3^Department of Psychology, University of Arizona, Tucson, AZ, USA; ^4^David Geffen School of Medicine at UCLA, Los Angeles, CA, USA

## Abstract

Physical activity improves overall health and reduces the risk of many negative health outcomes and may be effective in improving cognition, independent functioning, and psychological health in older adults. Given the evidence linking physical activity with improvements in various aspects of health and functioning, interventions exploring pathways for decreasing risk of dementia in those with mild cognitive impairment (MCI) and improving outcomes for those with dementia are of critical importance. The present review highlights the work examining physical activity interventions in order to achieve a comprehensive understanding of the potential benefits of physical activity for individuals experiencing cognitive decline. The primary focus is on aerobic exercise as this is the main intervention in the literature. Our review supports the thesis that physical activity can promote healthy aging in terms of cognition, independent functioning, and psychological health for individuals experiencing cognitive decline. Specifically, physical activity improves cognition, especially executive functioning and memory in MCI, independent functioning in MCI and dementia, and psychological health in dementia. Given that benefits of physical activity have been observed across these domains, such interventions provide an avenue for preventing decline and/or mitigating impairment across several domains of functioning in older adults with MCI or dementia and may be recommended (and adjusted) for patients across a range of settings, including medical and mental health settings. Further implications for clinical intervention and future directions for research are discussed.

## 1. Introduction

The existing literature on physical activity in patients with MCI and dementia highlights improvements in physical health, especially aerobic health/fitness, as a crucial factor in improving brain health ([1]; for a review, see [2]). Physical activity improves overall health and reduces the risk of many negative health outcomes, including coronary heart disease, stroke, certain cancers, type 2 diabetes, obesity, hypertension, osteoporosis, falls, and mortality [3–6]. Although older adults (typically defined as age ≥ 65 years old) have the highest rates of the illnesses mentioned above, they are the least physically active age group and spend a significant proportion of their day being sedentary [6]. Physical activity also may be effective in improving cognition, independent functioning, and psychological health [4, 7, 8]. As such, randomized controlled trials (RCTs) have been conducted in order to carefully test the impact of physical health on cognitive, daily, and psychological functioning. The majority of research on physical activity in older adults has focused on healthy, community-dwelling individuals, yet there is promising evidence emerging regarding the benefits of physical activity even for frail and/or cognitively impaired older adults [9]. Therefore, physical activity should be explored as a potential pathway for decreasing risk of dementia in those with mild cognitive impairment (MCI)—individuals at increased risk for developing dementia—and improving outcomes for those already diagnosed with dementia.

Previous studies investigating the effects of physical activity among those with MCI or dementia have generally focused on outcomes in specific domains of functioning, such as cognition [10] or independent functioning [11]. Such domains are often distinct but interrelated [12], and there is some evidence that physical activity can support several domains of functioning concurrently, including cognition, independence in daily activities, and psychological health/quality of life [13]. Hence, the present review is aimed at highlighting the work in each of these areas in order to support a more comprehensive understanding of how physical activity may benefit individuals experiencing cognitive decline. Physical activity interventions targeting older adults have included a wide variety of programs, including aerobic exercise (e.g., walking), resistance training, yoga, and tai chi, and there is some support for each of these forms of activity having a positive impact on healthy aging (e.g., [14–16]). We decided to use the more inclusive term, physical activity, given the diversity of interventions included in the literature; however, the most common intervention type was aerobic exercise. Thus, the goal of this literature review is to characterize the utility of physical activity, especially aerobic exercise, in preventing or lessening the impact of declines in cognition, daily functioning, and psychological health among individuals with MCI and dementia. To this end, we searched online repositories (i.e., PubMed, PsycINFO) using the following primary search terms: (“mild cognitive impairment” or “neurocognitive disorder” or “dementia”) and (“physical activity” or “exercise”), combined with terms related to cognitive/neuropsychological assessment, brain imaging, ADLs/IADLs/independent functioning, and mental health/quality of life/social connectedness. The present review focused on meta-analyses, particularly those published within the past 10 years, whenever available. As an aside, due to changes in the criteria for MCI, the following literature review uses the term MCI to refer to all four subgroups of MCI now diagnosed. Most of the literature reviewed was done in accord with previous criteria [17, 18] and as such is reflective of amnestic MCI patients, both single and multiple domains. When studies distinguished between amnestic and nonamnestic MCI, we have indicated this; however, such instances were rare. Moreover, most studies did not incorporate biomarkers into the diagnostic procedures, and thus, specification of MCI due to AD was not used.

## 2. Cognition in Older Adults

Better physical health, especially cardiovascular health, contributes to individual differences in cognitive functioning in several cognitive domains, including executive functioning and memory in general populations of older adults [19]. Executive functions are sets of cognitive processes used in complex tasks, such as planning, strategizing, decision-making, updating working memory, inhibition of irrelevant information, and switching flexibly between two tasks [20, 21]. Cross-sectional and prospective studies have demonstrated that individuals who are more physically active show improvements in neurocognitive functioning compared to more sedentary individuals [22, 23]. However, two observational, prospective studies in a sample of older adults, Verghese et al. [24, 25], did not find associations between physical leisure activity and lower risk of developing either amnestic or nonamnestic MCI, respectively. This null finding may be at least partially explained by the forms of physical activity included, such as tennis, golf, climbing more than 2 flights of stairs, and babysitting, given evidence of a dose response with higher intensity of physical activity being associated with better cognitive functioning in older adults [26]. Further, there is evidence from longitudinal studies that physical activity may prevent cognitive decline and dementia and that people with higher levels of physical activity are at reduced risk compared to those with lower levels [27].

### 2.1. Physical Activity Interventions and Cognition among Those with MCI

Intervention efforts, such as those aimed at reducing cardiovascular risk factors and preventing stroke by increasing physical activity, have been proposed as effective methods for preventing further cognitive decline among those with MCI [28]. For example, in a sample of older adults with cognitive impairment but no diagnosis of dementia, Blumenthal et al. [29] observed modest, yet significant, cognitive gains in global cognitive functioning (*d* = .36) and executive function (*d* = .32) following participation in physical activity intervention, but no significant gains were observed for memory (*d* = .19) or language (*d* = .12). Similarly, Lautenschlager et al. [30] found that a 6-month physical activity intervention led to significant, yet modest, improvement across cognitive domains for older adults at risk for Alzheimer's disease. A meta-analysis of aerobic exercise intervention studies, which attempted to address shortcomings of individual studies (i.e., small sample sizes and effect sizes), demonstrated that improved fitness also improves cognitive function, especially in the domain of executive functioning [4]. More recently, a systematic review of RCTs spanning the adult lifespan revealed modest improvements in the domains of attention and processing speed, executive functioning, and memory following aerobic exercise interventions [10]. Importantly, those with MCI had smaller benefits (albeit benefits) in the domain of executive functioning but had somewhat larger gains in memory than cognitively healthy individuals. Regarding working memory, older samples, but not younger samples, benefitted from physical activity; the authors were unable to test for differences based on MCI status. Finally, physical activity benefitted attention and processing speed in a uniform manner that did not differ based on sample ages or MCI status.

A systematic review of RCTs specific to cognitively impaired individuals found that participants with MCI demonstrated improved global cognition, executive functioning, attention, and memory with increased physical activity [31]. Most studies included in Öhman et al. [31] did not distinguish between MCI subtypes (i.e., amnestic versus nonamnestic). One exception was Suzuki et al.'s [32] physical activity randomized control intervention, which included groups of both amnestic and nonamnestic MCI subtypes. These authors found that across MCI subtypes, their physical activity intervention was not associated with better maintenance of cognitive functions relative to controls. However, when examining the amnestic MCI group in isolation, those who participated in the physical activity intervention had improved immediate memory from baseline to end of intervention at 6 months compared to controls. Further, within the amnestic MCI group, controls showed declines in global cognition and had significant whole-brain cortical atrophy over the 6-month period whereas those who participated in the intervention did not evidence such negative outcomes over time. Taken together, Suzuki et al.'s [32] findings suggest that those with amnestic subtype MCI may uniquely benefit from physical activity interventions.

### 2.2. Physical Activity Interventions and Cognition among Those with Dementia

Regarding those already diagnosed with dementia, there is mixed evidence of physical activity improving cognition. One of the most cited meta-analyses on the topic [33] found that those enrolled in physical activity interventions showed greater improvements in cognition relative to controls. However, more recent meta-analyses have found lesser or no benefits. For example, Forbes et al. [34] reported a questionable benefit of physical activity intervention on cognition among those with dementia, which was qualified due to sizable heterogeneity between studies. Further, in Forbes et al.'s [35] most recent meta-analysis, they failed to detect a benefit of physical activity intervention on cognitive functioning across nine randomized clinical trials with over 400 participants. Similarly, Öhman et al.'s [31] review determined that physical activity interventions did not evidence clear overarching benefits on cognition, which the authors suggest may be due to methodological issues (e.g., poorly defined criteria for determining dementia status). Alternatively, for those with dementia, overall lifestyle may be more predictive of cognitive gains than a time-limited physical activity intervention [36]. Perhaps the level of neurodegeneration in dementia makes cognitive gains more difficult to achieve, and other outcomes (e.g., increases in independent functioning and psychological health) should be targeted in physical activity interventions. More research is needed to examine how to maximize the benefits of physical activity across varying levels of cognitive functioning.

### 2.3. Possible Mechanisms

Across individuals of varying cognitive functioning, the benefits of physical activity on cognition are thought to be mediated by various brain mechanisms [37]. One pathway by which physical activity impacts the brain is via the prevention or better management of cardiovascular risk factors (e.g., diabetes, hypertension, hyperlipidemia, and obesity). In fact, among those with MCI, presence of cardiovascular risk factors is associated with increased likelihood of progression to dementia [38]. Cardiovascular risk factors are associated with various changes to brain health. For example, hypertension is associated with hardening of the blood vessels to the brain, which increases risk for stroke [39], and can lead to small vessel damage in the form of white matter hyperintensities seen on magnetic resonance imaging [40, 41]. Obesity is associated with increased inflammation to the brain [42, 43] and has been associated with reduced cerebral blood flow [44, 45]. In addition to improving brain health by reducing cardiovascular risk factor burden, physical activity is thought to improve brain health by increasing neurogenesis and synaptic plasticity (for a review, see [37]). Physical activity, especially aerobic exercise, is associated with increases in brain-derived neurotrophic factor (BDNF), a serum that stimulates cell growth and maintains neurons [46]. Even acutely, physical activity is associated with increases in BDNF (measured in the periphery), though these immediate effects may be specific to males, not females [47]. Of note, physical activity is better at immediately increasing BDNF serum levels compared to cognitive training and meditation [48], evidencing a distinct mechanism by which physical activity enhances brain function. Further, the benefits of physical activity interventions on executive functioning may be mediated by increases in BDNF, especially among older adults [49].

Cognitive neuroscience studies employing neuroimaging techniques provide additional evidence for the impact of physical activity on brain function and structure. Functional connectivity, the extent to which different regions of the brain activate in sync with one another either during a task or when one is not directed toward a specific task (also known as resting state), is one measure of brain function. Increased functional connectivity in the default mode network (DMN), a commonly studied activation network that is hypothesized to be important for introspection and memory retrieval [50], during resting state was observed in older adults with MCI following 12 weeks of aerobic physical activity [51]. Changes to DMN connectivity are found in many diseases of aging [52–54]. Functional connectivity changes have also been observed in different brain networks following other forms of physical activity training [55, 56], suggesting specificity of effects dependent on the form of physical activity training. Somewhat surprisingly, among women with MCI who completed a resistance training, and not those who completed an aerobic exercise intervention, increased region-specific brain activity during an associative memory task was linked to better performance on the task.

In addition to using neuroimaging metrics to understand functional brain changes that occur following interventions, researchers have examined general relations between physical activity and brain structure. Physical activity may be a good predictor of long-term changes to brain structure, such as brain volumes, and in-turn risk for dementia, especially for those who average more physical activity than their peers [57]. Regarding training, participation in an aerobic exercise intervention was found to have increased BDNF serum levels and increased volume of the anterior hippocampus, and this was associated with improvements in spatial memory among older adults without cognitive impairment [58]. With regard to physical activity interventions, better aerobic health/fitness was associated with better white matter integrity (a measure of the health of white matter microstructure) only among those who completed aerobic exercises but not among those who completed stretching exercises [59]. Although better white matter integrity is typically associated with better cognitive functioning, the authors did not detect such relationships [59].

There is growing evidence suggesting that physical activity helps maintain brain health among those at risk for Alzheimer's disease. Specifically, among at-risk individuals, greater physical activity is associated with fewer changes to the brain that indicate preclinical Alzheimer's disease (e.g., reduction in hippocampal volumes and increased *β*-amyloid burden; [60]). Further, among cognitively healthy late-middle-aged adults with family history of and/or genetic risk for Alzheimer's disease, moderate, rather than light or vigorous, physical activity may be most beneficial to maintaining brain function as measured by glucose metabolism [61]. Although there is evidence for physical activity improving and maintaining brain health among cognitively healthy older adults and those with MCI, which may then confer benefits on cognition, these relations are not well understood among those with established dementia.

## 3. Independent Function

The impact of physical activity interventions on functional independence largely seems to be beneficial. In a national longitudinal study that included Americans across the age span, Cotter and Lachman [62] found that several factors, including greater physical activity, were associated with better self-rated physical health and less physical disability 9 years later. Moreover, greater physical activity and smaller waist circumference attenuated age-related increases in physical disability. Likewise, an observational study of community-dwelling older adults with functional limitations found that health behaviors, particularly physical activity, contributed to functional independence [63]. In contrast, Gu and Conn's [11] meta-analysis of physical activity interventions in older adults did not reveal benefits on participants' activities of daily living, but the authors found that physical activity interventions improved functional performance across a variety of tasks that were designed to simulate activities of daily living. In their 12-year longitudinal study of older adults without baseline activity limitations, Rist et al. [64] demonstrated that among individuals with high and low probability of dementia, physical activity was associated with decreased odds of developing limitations to instrumental activities of daily living (everyday activities that are more cognitively involved than basic activities of daily living). In contrast, physical activity did not appear to be protective of basic activities of daily living [65], suggesting that physical activity may have specific protections for maintenance of more cognitively demanding tasks.

### 3.1. Physical Activity Interventions and Functioning among Those with Dementia

In McLaren et al.'s [66] systematic review of nonpharmacological RCTs for community-dwelling individuals with dementia, all six RCTs that examined physical activity reported statistically significant improvement in functional ability among those in the physical activity intervention groups. Forbes et al. [34, 35] have consistently detected benefits of physical activity on daily functioning though they note that there is significant heterogeneity between randomized control trials. Blankevoort et al.'s [67] review provided evidence that compared to resistance training alone, multicomponent physical activity interventions benefitted physical functioning and functional independence in all stages of dementia. Further, the greater the physical activity volume during the intervention (as measured by number of sessions and time per session), the greater the functional improvements [67]. Zeng et al. [68] also found that physical activity improved physical functions such as reach, balance, and mobility, which are core parts of basic activities of daily living (e.g., toileting).

Physical activity interventions in long-term care settings also have varied effects. For example, in a study of patients with mild-to-moderate dementia who were living in residential care facilities, a high-intensity physical activity program slowed decline in functional independence and improved balance, but only in patients with non-Alzheimer's-type dementia [69]. Another physical activity intervention for patients in an acute psychiatric ward, on the other hand, delayed loss of mobility in patients with moderate-to-severe dementia but did not significantly impact other forms of daily living [70]. Thus, more research is needed to clarify the extent to which physical activity may affect functioning in patients who are institutionalized.

### 3.2. Additional Considerations

It is important to consider the role of cognition when measuring functional independence outcomes. Cognition and functional independence impact one another with declines in one often predicting declines in the other; most often, cognitive declines precede and predict functional declines [12]. Given this relationship, it is crucial to understand how to promote independence in older adults experiencing cognitive decline. Oftentimes, approaches like Poulos et al.'s [71] comprehensive “reablement” approach for those with mild-to-moderate dementia include both pharmacological and nonpharmacological approaches to support daily functioning, such as goal-based cognitive rehabilitation and physical activity, targeted rehabilitation following acute illness/injury, and caregiver education and support. Consistent with this model, health behaviors, especially physical activity, have been implicated in older adults' abilities to maintain functional independence [62, 63]. Thom and Clare [72] highlighted evidence that physical activity and cognitive rehabilitation, independently, have been shown to improve functioning in older adults and, to some degree, those diagnosed with dementia. Further, they suggested that the combination of these types of interventions may enhance improvements in functional independence in those with dementia because they could target both physical and cognitive deficits relevant to preventing functional dependence, including, for example, cardiovascular risk and risk of falls with physical activity, and behavioral and problem-solving strategies with cognitive rehabilitation, thereby maximizing efficacy of intervention efforts above and beyond the effects of a single-domain intervention [72]. Others posit that physical activity may impact functional independence by improving cognitive domains that may be especially crucial to maintaining independence, such as executive functioning [73].

Another important consideration to these relationships, especially in individuals with dementia, is involvement of families, friends, and other social supports, particularly those with caregiving responsibilities. The availability of caregivers provides greater opportunity for the person with dementia to remain more functional and engaged in meaningful activities in their communities [74]. However, caregiver burden, stress, depression, anxiety, poor health, social isolation, and financial hardship all can negatively affect caregivers and, indirectly, the person with dementia as well [75]. In addition to promoting the functional independence of people with dementia, supporting caregivers directly, especially targeting their psychological well-being and providing information, can benefit both the caregiver and the person with dementia [75, 76]. Often, resources such as time and transportation limit the accessibility of interventions. Working with caregivers, Vreugdenhil et al. [77] implemented a community-based home physical activity program that involved patients with Alzheimer's disease walking under the supervision of their informal caregivers. The intervention was effective at improving patients' cognition, mobility, and instrumental activities of daily living, highlighting the potential gains of working with caregivers; of note, however, is that no caregiver outcomes were included in this study. When informal caregivers are unable to provide support for a person with dementia, long-term care placement becomes an alternative solution. In a meta-analysis of nursing home placements of older adults with dementia, impairments in functional independence, poorer cognition, and behavioral/psychological dysfunction were significant risk factors for placement in long-term care [78]. These authors also found an association between caregiver burden and risk of institutionalization and recommended caregiver education and support to delay placement in nursing homes. Therefore, it is vital to identify ways to maintain independence among those with cognitive decline.

## 4. Psychological Health

Research on the effects of increased physical activity on emotional and social functioning also has been an important area of study. Meta-analyses have demonstrated that physical activity interventions reduce symptoms of depression and anxiety in healthy adults, including older adults [79, 80]. Physical health is linked to decreased depression and loneliness and improved mood and life satisfaction [81].

### 4.1. Physical Activity Interventions and Psychological Health among Those with Dementia

Among older adults with dementia, physical activity interventions improved general psychological well-being across a variety of neuropsychiatric symptoms (for a meta-analysis, see [68]). Meta-analytic studies have also found that physical activity is associated with improved mood and well-being [8, 82]. However, Forbes et al.'s [34, 35] meta-analyses examining the impact of physical activity interventions on depression among those with dementia did not find a benefit of intervention. Forbes et al. [34, 35] were also interested in examining quality of life outcomes but were unable to do so due to lack of information on the outcome in the studies they investigated. Older adults with dementia report lower quality of life, and there is preliminary evidence that certain interventions mitigate this effect [83]. For example, in a systematic review of different types of nonpharmacological interventions, Cooper et al. [83] noted that factors, such as improved caregiver coping and whether someone lives at home versus in a nursing facility, may influence the impact of interventions on improving quality of life for people with dementia. Taken together, this emphasizes the need for more research regarding mediators, moderators, and individual differences in interventions aimed at improving quality of life. Regarding physical activity interventions, increased physical activity was found to improve quality of life indirectly, by first reducing distress among older adults [84]. It is unclear if such a relationship would differ in those with MCI or dementia. Interestingly, brain integrity, which tends to be poorer in those with cognitive impairment, may be a good predictor of mental health improvements following physical activity. In a large sample of older adults, larger amygdala volumes predicted greater decreases in loneliness following physical activity intervention [85]. In addition, larger prefrontal cortex volume predicted greater reductions in stress following physical activity. Regarding mechanisms, BDNF (a promoter of neuronal health and function) levels are lower in individuals with psychological dysfunction (e.g., in depression), and increases in BDNF via physical activity may improve functioning in brain regions/circuits key in maintaining psychological well-being (e.g., in the frontal cortex and hippocampus; [86]).

Alternatively, psychosocial health may impact physical activity. Social connectedness, or the degree to which an individual engages in social interactions and activities of societal value, is an additional construct that is relevant to older adults' health and well-being. Levels of social connectedness have been associated with better health and successful aging [87, 88]. However, the study of this construct in relation to physical activity is less prominent in the literature, and there are conflicting findings in the few studies that do include measures of this construct. Some studies have demonstrated that people with more active social lives have better quality of life and physical/health function [87, 89]. However, the patterns by which all three components—physical activity, social connectedness, and cognitive functioning—interrelate are unclear [90, 91]. Participants in physical activity programs also have identified social connectedness as an attraction to engaging in such interventions [92]. However, a large, longitudinal study found no evidence of an association between social connectedness and successful aging [93]. These authors suggested that social connection may be related to self-perceptions of successful aging but does not actually lead to improved outcomes in the aging process. It will be important to continue studying the interrelations between physical activity and psychological functioning, particularly in the context of cognitive decline and functional dependence, as this literature has not yet clarified such pathways. The interrelations among these domains are key when determining how to optimally approach rehabilitation for adults with MCI and dementia.

## 5. Conclusions

While promotion of physical activity is encouraged throughout the lifespan, it can have particularly important effects on older adults, especially those experiencing cognitive impairment. In this review, we described the benefits that physical activity can have on individuals' functioning across several domains, including cognition, functional independence, and psychological health. Specifically, physical activity improves cognition, especially executive functioning and memory in MCI, independent functioning in MCI and dementia, and psychological health in dementia. Often, the effect of physical activity on each of these functional domains has been studied separately and in diverse samples at various points in aging or cognitive/functional decline. Considering these findings all together, it becomes clear that increased physical activity has the potential to improve functioning across several important domains in older adults at risk for or diagnosed with MCI or dementia. Therefore, future research clarifying the effects of exercise on these domains concurrently, as well as the interrelations among these constructs, is likely to provide useful information regarding optimal approaches toward prevention and/or rehabilitation efforts for older adults with MCI or dementia.

Clinically, physical activity may be considered an important part of health promotion in medical settings, when not contraindicated for a given patient. This is true even in frail older adults and/or individuals with cognitive impairment. Given that benefits of physical activity have been observed across domains of cognition, independent functioning, and psychological health, such interventions may be recommended (and adjusted) for patients across a range of settings, including medical and mental health settings. Much of the existing literature has supported aerobic exercise interventions as promoting cardiovascular and brain health, as well as other forms of healthy aging. However, few studies have directly compared the effects of different forms of physical activity (e.g., aerobic exercise vs. resistance training, aerobic exercise vs. yoga, and aerobic exercise vs. balance training) on multiple domains of aging, and this will be an important area for future research to explore. The ideal format or amount of physical activity in order to maximize efficacy is unclear. The “amount” (e.g., frequency, duration) of physical activity varies considerably across studies, and future research will need to determine the optimal amount (or ideal range) of physical activity to maximize benefits in a given population. The World Health Organization recommends that older adults complete a minimum of 150 minutes of moderate-intensity aerobic physical activity, 75 minutes of vigorous-intensity aerobic physical activity, or an equivalent combination of moderate- and vigorous-intensity activity each week [94]. While these recommendations are based on research, more structured protocols should be developed and examined in order to determine the optimal amount of physical activity for older adults.

There are several knowledge gaps in the literature that represent important limitations of the present review and suggest potential avenues for future research. First, as mentioned previously, the current state of the literature generally includes studies with small sample sizes. Larger sample sizes are needed to determine the effect sizes of various results. Moreover, when examining effects on multiple cognitive domains, parts of the brain, and/or symptoms and behaviors, larger sample sizes are needed to determine who benefit most from what exact interventions. In addition, there are varied protocols with different forms of physical activity of varying frequency and duration, and therefore, it is difficult to compare or consolidate results from individual studies. Meta-analyses, which were the focus of this review, when available, counteract some of these shortcomings (e.g., small sample sizes, small effect sizes), but they cannot tease apart the effects that such characteristics pose. In addition, the vast majority of the research on physical activity in older adults with cognitive impairment involves individuals diagnosed with MCI and Alzheimer's disease, with fewer studies focused on other forms of dementia, such as cerebrovascular or Lewy body dementias. We also did not find many studies that mention the issue of behavioral disturbance in dementia, as this factor is generally not mentioned or may even be an exclusionary criterion. In our literature review, we decided not to include studies specifically studying individuals with Parkinson's disease, which has a large separate literature on physical activity of its own.

Additional limitations of the literature, and therefore, our review, are lack of information on intrapersonal and extrapersonal mediators between physical activity and cognition, functional independence, and psychological health. Studies that are sufficiently powered to examine longitudinal interactions and mechanisms are required. In addition, it is important that future studies include measures from multiple domains concurrently, including physical, cognitive, neuroimaging, functional, and psychological outcomes, following physical activity interventions. These effects will be particularly important to examine longitudinally and in relation to one another so that the specific course and therefore mechanisms may be illuminated. One final point is that cost-effectiveness should be considered in future research so as to determine the optimal and most efficient interventions in the promotion of healthy aging and specifically prevention and rehabilitation of MCI and dementia.

Overall, the present review summarizes the current state of the literature that exists on the effects of physical activity on cognition, independent functioning, and psychological health, all of which are important aspects of healthy aging. We found strong evidence that physical activity interventions provide an avenue for preventing decline and/or mitigating impairment across several domains of functioning in older adults with MCI or dementia.
